# Multifold Enhanced Raman Detection of Organic Molecules as Environmental Water Pollutants

**DOI:** 10.3390/bios13010004

**Published:** 2022-12-21

**Authors:** Yunyun Mu, Miao Liu, Jiajun Li, Xinping Zhang

**Affiliations:** Institute of Information Photonics Technology, Faculty of Science, Beijing University of Technology, Beijing 100124, China

**Keywords:** SERS hollow fibers, water pollution, toluene, trace detection, direct in-situ detection of environmental water

## Abstract

Organic molecules, including the benzene series, have been identified as pollutants in environmental water. Due to their very low solubility, they have very small concentrations in water, and they are difficult to be detected by conventional techniques. In particular, there is a lack of real-time, accurate, and rapid detection methods for such molecules in water. However, they are detrimental to human health in many aspects. Toluene has been an important indicator of such environmental pollution detections. In this work, we propose a 3D SERS scheme consisting of a hollow fiber that is coated on the inner wall with densely arranged silver nanoparticles, which supplies multifold Raman enhancement by the plasmonic microcavity. Strong confinement of excitation laser energy and strongly enhanced Raman signals with the bidirectional collection are utilized to achieve high-sensitivity detection of toluene molecules in water. Raman signal with a reasonable signal-to-noise ratio has been measured for a concentration of 0.53 mg/L, indicating a detection limit even lower than this value for such a Raman spectroscopic technique. The corresponding enhancement factor is higher than 6 × 10^3^ with respect to the available systems. Thus, this device not only enables direct trace detection and real-time monitoring of the water-polluting status by organic molecules but also supplies a practical approach for biological sensing.

## 1. Introduction

Detection of pollutant molecules in environmental water is a big challenge for environmental science since there are not yet effective and efficient approaches for achieving specific identification of different toxic organics in water with high sensitivity. So far, the content of organic substances is evaluated by the assessment of the total oxygen demand (TOD) in the biological oxidation process, which reduces the sensitivity and specificity of the testing technique. The benzene series or BTEX, including benzene, toluene, ethylbenzene, xylene, and chlorobenzene, are particularly detrimental to the human blood, nervous, and reproductive systems. Through the food chain, the benzene series can induce leukemia, damage the liver, and cause skin irritation and rashes [1,2,3,4,5,6]. Therefore, the determination of the benzene series’ existence and their concentration in environmental water is indispensable for controlling the quality of environmental water.

Toluene has always been an important indicator of environmental water pollution. Methods for toluene removal [7,8], degradation [9,10,11,12,13,14], and adsorption degradation [15,16,17] have been reported. Testing of toluene in the forms of vapors [18,19,20,21,22,23] has been demonstrated in different methods. However, the detection of the toluene molecules in water has rarely been reported using Raman spectroscopy. This is mainly because of the indissolvableness of toluene in water, which has a concentration of only 530 mg/L for the saturated aqueous solution at 20~25 °C. Furthermore, such a low concentration is not detectable by the conventional Raman spectroscopic methods. However, reliable justification of the existence of such molecules in domestic water without exceeding the safety line is very important not only for human life but also for controlling industrial discharge. Capillary and optical fibers [24,25] have been used for liquid sensing; however, there is still a lack of effective methods for the direct detection of organic molecules like toluene in water.

In this work, we demonstrate a SERS hollow fiber approach for the direct detection of toluene molecules in water, where no preprocessing of the water species and no enrichment of the target molecules are required for such a detection technique. Multifold enhancement mechanisms for the Raman signals are supplied in such a microcavity scheme, and the bidirectional collection design enables further improvements of the signal-to-noise ratio so that we have achieved the best result so far for the direct detection of BTEX molecules in water.

## 2. Design of Raman Detection Scheme by Plasmonic Hollow Fibers 

**Figure 1:**
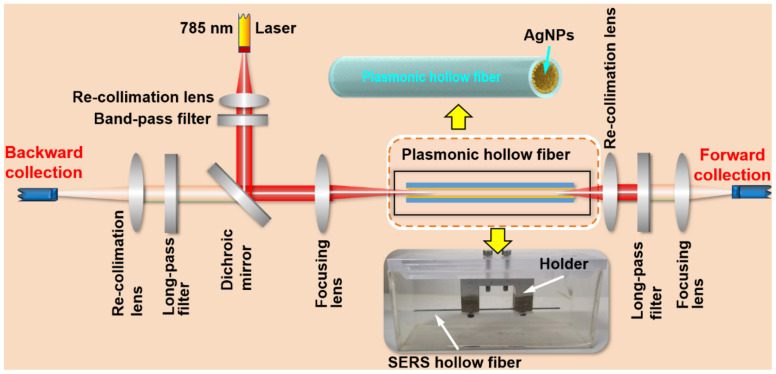
Design of the multifold enhanced Raman detection system.

Figure 1 shows the Raman scheme with multifold enhancement mechanisms, which is designed specifically for the detection of low-concentration molecules in water. This is a home-built Raman system, where a fiber-coupled 785 nm laser is first recollimated before passing through a 785-nm band-pass filter and reflected by a dichroic mirror. The excitation laser is then focused by a lens with a focal length of 30 mm into the SERS hollow fiber. The back-scattered Raman signal is recollimated by the focusing lens, passes through the dichroic mirror and a long-pass filter, and is focused again into the detection head of the fiber spectrometer. Meanwhile, the forward-scattered Raman signal is also recollimated by a lens, passes through a long-pass filter, and is focused on the detection head of the fiber spectrometer. These two signals are added together to produce the final detection signal. In all of the measurements using this system, we used an integration time of 60 s.

The SERS hollow fiber is fixed by a metal holder and immersed the sample water in a quartz cuvette with dimensions of 10 × 5 × 5 cm^3^, where the hollow fiber has a length of 10 cm and an inner diameter of 400 μm, as shown by the photograph indicated by the downward yellow arrow. The inner wall of the hollow fiber is coated homogeneously with silver nanoparticles, as illustrated in the drawing indicated by the upward yellow arrow. The silver nanoparticles are chemically synthesized and dispersed in xylene to produce the colloidal solution with a concentration of 100 mg/mL. Then, the colloidal solution is flowed through the hollow glass fiber multiple times to coat a layer of silver nanoparticles onto the inner wall of the hollow fiber [26]. Then, direct laser writing is employed to produce the SERS inner wall, where the corresponding method is described in detail in Ref. [27]. To ensure that the water sample in the cuvette can fill the SERS hollow fiber completely, the inner surface of the hollow fiber is processed in advance by flowing 3-Mercaptopropionic acid (3-MPA) through the hollow so that the inner-wall surface becomes strongly hydrophilic.

## 3. Multifold Enhancement Mechanisms

**Figure 2:**
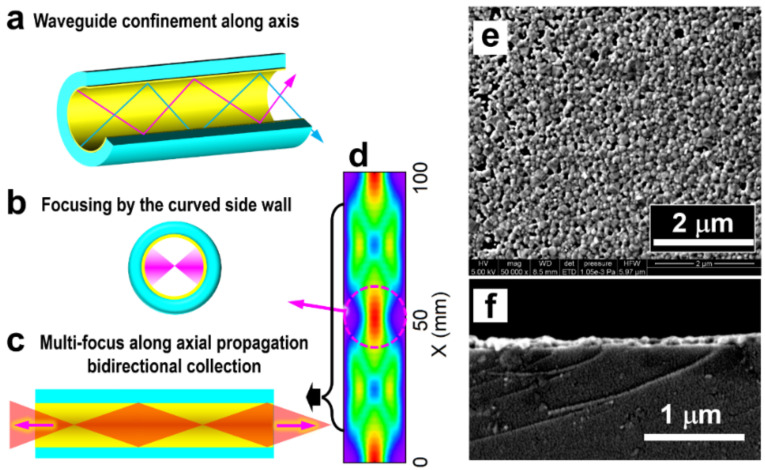
Multifold optical feedback and enhancement mechanisms. (**a**) Confinement of the excitation and Raman light beams by the microcavity formed by the AgNP-coated inner wall of the hollow fiber. (**b**) Focusing the reflected light beam by the curved surface of the inner wall. (**c**) Bidirectional collection of the Raman scattering light. (**d**) Simulation of the optical electric field distribution in the hollow fiber. (**e**,**f**) SEM images were measured on the top surface and the cross-sectional profile of the Ag-coating on the inner wall of the hollow fiber.

Figure 2 demonstrates multiple enhancement mechanisms for the Raman interactions in the hollow fiber with coatings of Ag nanoparticles. These mechanisms integrate optical feedback, bidirectional signal collection, and the SERS effects. Although they play different roles at different locations for total enhancement, their complementary effects make the plasmonic hollow fiber an advantageous scheme for the direct detection of low-concentration molecules in liquids.

### 3.1. Optical Feedback by Metallic Inner Wall

The silver coating with high optical reflection on the inner wall of the hollow produces both a waveguide and a microcavity. As shown in Figure 2a, both the excitation and the Raman scattering light in the meridional planes and sagittal planes are highly reflected and confined in the hollow space during propagation along the fiber. This enhances the interaction by tightly confining the excitation light energy and extending the interaction distance largely. Meanwhile, the curved internal surface supplies light-focusing mechanisms so that the focus of the excitation laser can be reproduced in the liquid body center area of the hollow space, as shown in Figure 2b, which enables stronger Raman interaction. Moreover, the excitation laser beam is coupled to the hollow fiber after being focused near the entrance at one end of the hollow, as shown in Figure 2c. Therefore, the focusing effect is repeated along the propagation direction, implying multiple focuses of the excitation laser beam and producing multifold optical feedback and enhancement processes.

**Figure 3:**
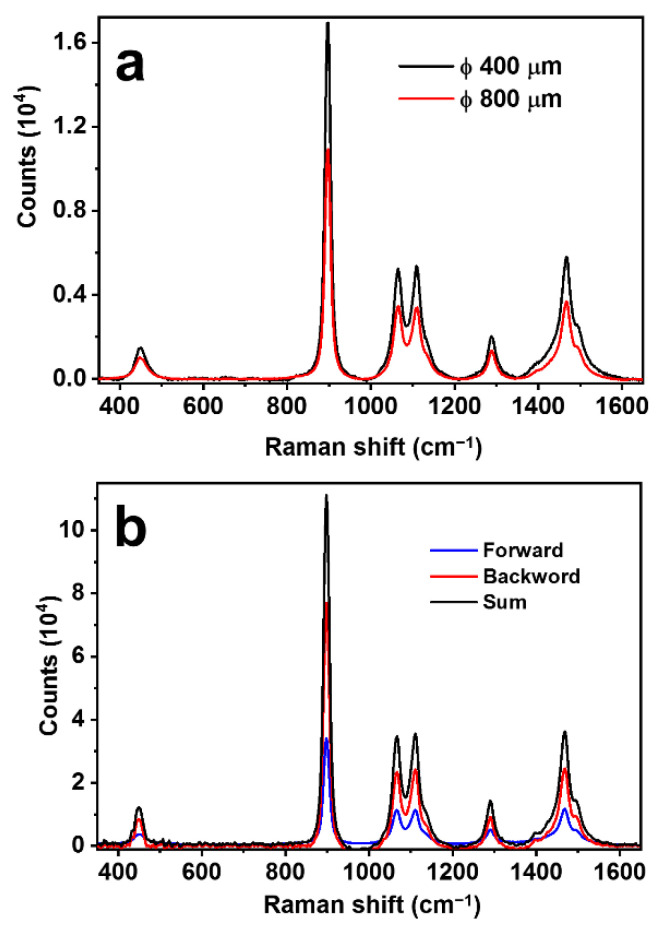
(**a**) Raman spectra measured on pure ethanol using SERS hollow fibers with diameters of 400 and 800 μm. Employed parameters: focal length = 30 mm, excitation laser power = 200 mW, integration time = 1 s. (**b**) Raman spectra were measured on pure ethanol using a SERS hollow fiber with a diameter of 400 μm using different collection geometry: forward (blue), backward (red), and bidirectional (black). Employed parameters: focal length = 30 mm, excitation laser power = 450 mW, integration time = 1 s.

It is understandable that there is an optimized value of the diameter of the hollow fiber. Two factors should be considered: (1) Smaller diameters mean stronger confinement of the light beam and enhanced interactions. (2) Focusing of the light beams by a curved metal-coated surface requires suitable curvature so that the excitation laser beam with oblique incidence can be properly focused into the free space of the hollow. Apparently, the small inner diameter supplying stronger light confinement and stronger Raman interactions plays more important roles. This can be verified by the experimental results in Figure 3a, where a hollow diameter of 800 and 400 μm in the same measurement setup for the same conditions. Clearly, the 400 μm hollow fiber produces an enhancement of more than 55% than the 800 μm case. For safety and convenience, the measurement was carried out on pure ethanol.

### 3.2. Bidirectional Raman Detection Scheme

In most of the conventional Raman detection schemes, the scattering signal in the backward direction opposite to the excitation is collected, and those in other directions are simply discarded. This is also based on the consideration of the easy management of the optics and the suitable design for practical applications. As has been demonstrated in our previous publication [28], optical feedback by a curved reflection mirror on the other end of the optical excitation path may enhance the Raman signal by nearly six times. Certainly, the feedback is working on both the excitation laser beam and the Raman scattering light beam. This result convincingly confirms the existence of the forward propagating Raman signals and evaluates the loss of Raman signal collection in the traditional scheme employing only the back-scattering Raman signals. Thus, for our scheme using a SERS hollow fiber with a symmetrically cylindrical shape and a large length-diameter ratio, which is immersed completely in the sample solution, as shown in Figure 1, it is more advantageous to collect both the backward and forward Raman scattering signals, as illustrated in Figure 2c. To evaluate the signal contribution in forward propagation, we managed an experiment using pure ethanol as a sample for detection. The results are shown in Figure 3b, where a comparison is made between the pure backward and forward Raman scattering signals. Clearly, the backward scattering is much stronger than forward scattering and there is an increase of 49.4% in the total signal intensity. Figure 2d shows an example of the simulation of the optical electric field distribution in the hollow space of the fiber, showing the additional focus of the excitation laser beam in the center area and energy convergence to the inner wall at different locations. Parameters close to those used in the practical configuration are employed in the simulation. The simulation results verify the proposed models in Figure 2b,c, as indicated by the left-toward arrows.

### 3.3. SERS Effects

The silver-coated inner wall of the hollow fiber is, in fact, a curved SERS substrate, as shown in Figure 2e,f by the SEM images measured on the top surface and the cross-sectional profile of the AgNP coating layer. Densely arranged AgNPs with small gaps support not only a strong SERS effect but also a high reflection of both the excitation and Raman light beams, supporting the formation of the microcavity in the hollow. Therefore, different from the conventional planar SERS substrates, it consists of a cylinder shell with a small diameter to form a microcavity for the incident light beams, enabling optical feedback and energy intensification.

However, it is apparently understandable that the light energy confinement effect is even stronger than the pure SERS effect since the plasmonic field has an interaction distance smaller than 100 nm, which even requires the falling of the target molecules into the plasmonic gaps. These mechanisms largely reduce the SERS effect for the tiny amount of mobile molecules in water. Nevertheless, the SERS effect makes negligible contributions to the total detection signals. 

### 3.4. Extension of the Interaction Length

An additional advantage of the plasmonic hollow-fiber scheme is the large extension of the Raman interaction length. According to our experimental results, the enhancement of the Raman signal is approximately proportional to the length of the plasmonic hollow fiber for our experimental design. In Figure S1, we show the Raman measurement results on pure ethanol using plasmonic hollow fibers with lengths of 4, 6, and 10 cm. There is roughly a linear relationship between the intensity of the Raman signal and the fiber length, as illustrated by the plot in the inset of Figure S1. However, such a linear dependence is verified only for a length shorter than 10 cm, which is not yet a maximum or optimized value for the fiber length. In our case, the compactness of the system is also important for portable equipment for in-situ detection. Meanwhile, a straight hollow fiber without any bending is apparently more advantageous. These considerations explain why we have chosen a fiber length of 10 cm.

## 4. Detection of Toluene Molecules in Water

For preparing the solution of toluene in water with a concentration of 530 mg/L, which is a bit lower than that of the saturated solution at 28 °C, 24 μL toluene was first dispersed into 40 mL deionized water with sonication. Considering the density of toluene of 0.86 g/mL, the prepared solution has a concentration of 530 mg/mL. Then, diluted solutions with concentrations of 53 mg/L, 5.3 mg/L, and 0.53 mg/L were prepared. The rate of the dispersion process was slow to make sure that toluene was fully dissolved in water. The diluted solutions with different concentrations are injected into the SERS hollow fibers by the capillary force, which is incorporated into the measurement setup in Figure 3 for different measurements.

**Figure 4:**
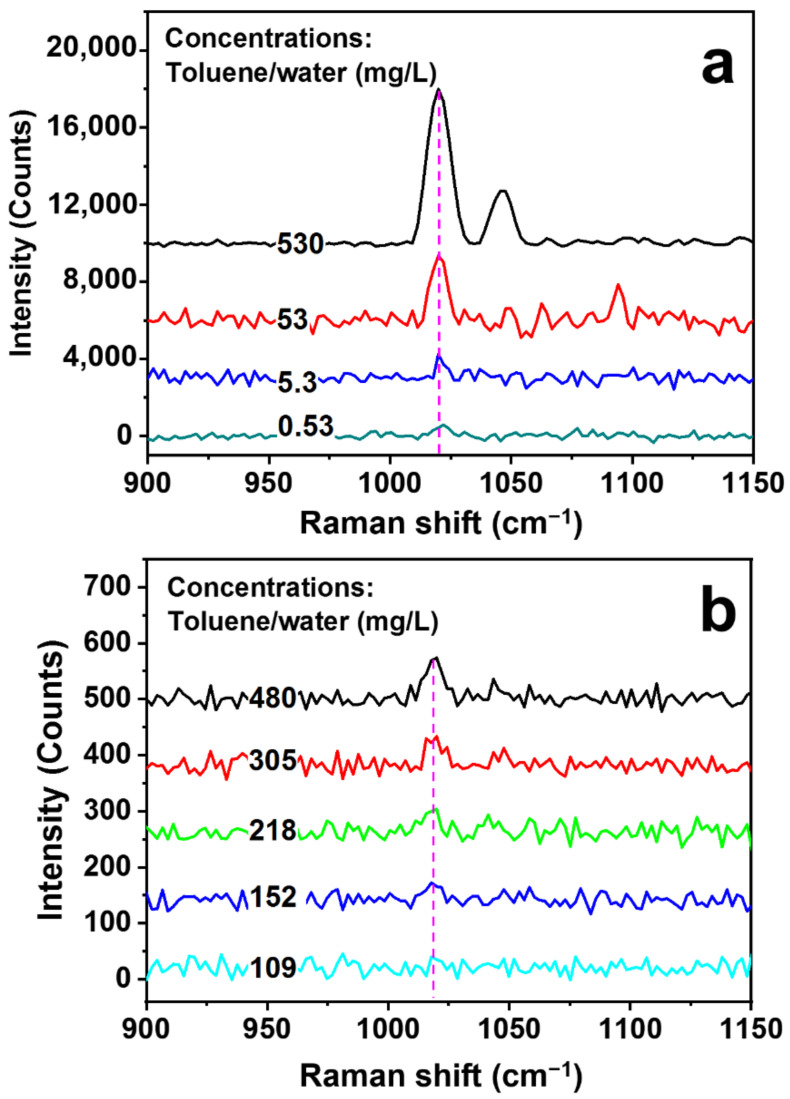
(**a**) Measurements of Raman spectra on the toluene/water solutions with concentrations of 530, 53, 5.3, and 0.53 mg/L using SERS hollow fibers. (**b**) Direct Raman measurement results of toluene/water solutions with different concentrations using a conventional scheme without SERS hollow fibers.

The specificity of toluene detection is ensured by the identification of its typical Raman peaks, as shown in Figure S2 by the measurement of pure toluene. The Raman peak at about 1020 cm^−1^ is the main signature of toluene, which is related to the in-plane C-H bending vibration. Therefore, this peak is employed as the main ruler for evaluating the Raman signals. Figure 4a shows the measurement results of the Raman spectra on the toluene/water solutions with concentrations of 530, 53, 5.3, and 0.53 mg/L using the SERS hollow fiber scheme in Figure 3. The variation of the signal amplitude as a function of the solution concentration is shown by the empty circles in Figure S3, which exhibit strong nonlinearity. According to the plot in Figure S3, even at a concentration as low as 0.53 mg/L (530 ppb), the Raman signal has a peak intensity of 517 counts, which is more than 15 times lower than that for a concentration of 530 mg/L.

As a comparison, direct Raman measurement results on toluene/water solutions with different concentrations are presented in Figure 4b, where the same laser, the same spectrometer, and the focusing lens with the same focal length as those in Figure 1 have been employed. The solution concentration is increased from 109 to 480 mg/mL, and nearly no Raman signal can be resolved for a concentration of 109 mg/mL. The plot of the Raman signal intensity as a function of the solution concentration is shown by the blue squares and in the inset of Figure S3. Even for the solution concentration of 480 mg/mL, a signal intensity as low as 70 counts has been obtained, implying much-reduced sensitivity, which is negligibly small as compared with that obtained for the SERS hollow fibers, as verified by Figure S3. The enhancement factor by the SERS hollow fiber with respect to the direct Raman detection scheme in Figure 1 can be evaluated by the measurement results in Figure 4, which is defined as:

(1) $$E_{f}=\frac{C_{D}\times S_{SHF}}{C_{SHF}\times S_{D}}$$

where *C_D_* and *C_SHF_* are the solution concentrations for the direct direction using a conventional Raman scheme and multifold enhanced detection using the SERS hollow fibers, respectively, and *S_D_* and *S_SHF_* are the corresponding signal intensities. Using the measurement results at about 1020 cm^−1^ for *C_SHF_* = 0.53 mg/L in Figure 4a and *C_D_* = 152 mg/L in Figure 4b, we may calculate an enhancement factor of *E_f_* = 6.17 × 10^3^.

Although our experimental results in Figure 4a justify a detection limit of 0.53 mg/L, the corresponding Raman signal at 1020 cm^−1^ still has good contrast. The direct signal-to-noise ratio (SNR), which is defined by I_S_/I_N_ with I_S_ and I_N_ denoting the signal and noise intensities, respectively, is measured to be larger than 1.7. This implies a possibly much lower detection limit than the observed value of 0.53 mg/L. In such a consideration, we plot the relationship between the SNR and the solution concentration using the measurement data, as shown in Figure S4 (open circles), and make a linear fitting (red line) to the measurement. If using the point at which the linear fitting line roughly reaches 1, we may obtain a detection-limit value of lower than 100 μg/L.

## 5. Conclusions

We report a high-sensitivity SERS sensing technique for toluene molecules in water and successful detection of a concentration lower than 0.53 mg/L (530 ppb), which is so far the best result for the direct detection of organic molecules in water and supplies an in-situ water pollution monitoring technique. A SERS hollow fiber is the central component of the sensing technique, which consists of AgNP-coating on the inner wall of a hollow glass fiber with an inner diameter of 400 μm and a length of 10 cm. Multiple Raman enhancement mechanisms are responsible for the sensing performance, which mainly include the microcavity effect with strong optical feedback and multifold focusing of the excitation laser beam, SERS effects by the AgNPs, the bidirectional collection of the Raman signals, the extension of the interaction distance by the long hollow space, and the tight confinement of the excitation light energy. Such a sensing technique with a multi-dimensional Raman enhancement configuration applies extensively to various water-pollutant organic molecules and is, therefore, of critical importance for environmental water quality testing, assessment, and protection. Furthermore, such a Raman scheme, as well as its related strategy and performance, is generally practical for the detection of various organic molecules with high sensitivity in aqueous solutions, including proteins or nucleic acids, in a biological environment with low concentrations.

## Data Availability

Not applicable.

## Supplementary Materials

The following supporting information can be downloaded at: https://www.mdpi.com/article/10.3390/bios13010004/s1, Figure S1: Raman signals measured on pure ethanol using plasmonic hollow fibers with different lengths; Figure S2: Raman spectrum measured on pure toluene; Figure S3: Raman signal intensity as a function of the toluene/water solution concentration measured using a SERS hollow fiber (empty circles, black) and measured directly using a conventional Raman scheme (empty squares, blue); Figure S4: Plot of the signal-to-noise ratio (SNR) of the measured Raman signal at 1020 cm^−1^ as a function of solution concentration and linear fitting of the measurement data.
