# Supplementary material for: Multifold Enhanced Raman Detection of Organic Molecules as Environmental Water Pollutants
Source: Biosensors (Basel). 2022 Dec 21;13(1):4. doi: 10.3390/bios13010004 (PMC9855845; doi:10.3390/bios13010004)
Supplement: Supplementary file 1 [file biosensors-13-00004-s001.zip › biosensors-2076018-supplementary.pdf]

Supporting Information

Multifold enhanced Raman detection of organic molecules as environmental water pollutants

Yunyun Mu, Miao Liu, Jiajun Li, and Xinping Zhang*

College of Physics and Optoelectronics, Faculty of Science, Beijing University of Technology, Beijing 100124, P. R. China

*Email: zhangxinping@bjut.edu.cn

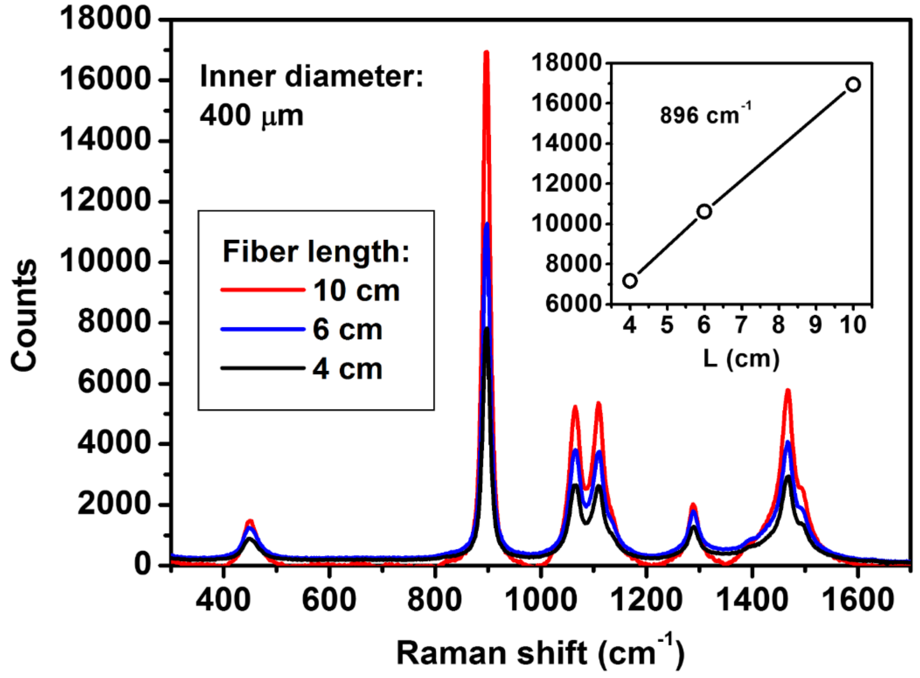

Fig. S1. Raman signals measured on pure ethanol using plasmonic hollow fibers with different lengths. Inset: Raman signal intensity at 896 cm^{-1} as a function of the length of the hollow fiber, showing a linear dependence.

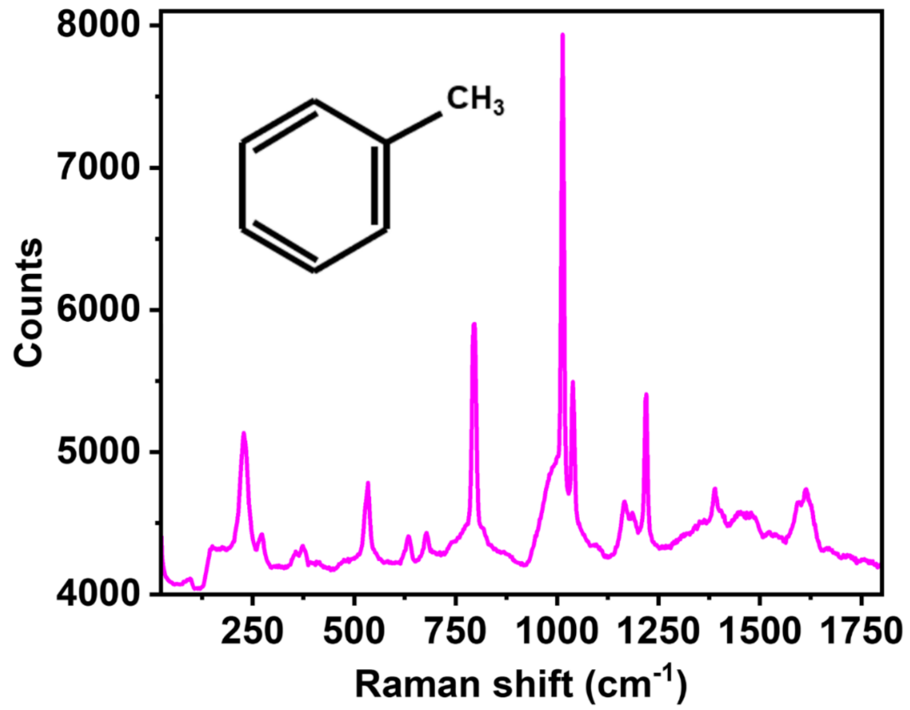

Fig. S2. Raman spectrum measured on pure toluene.

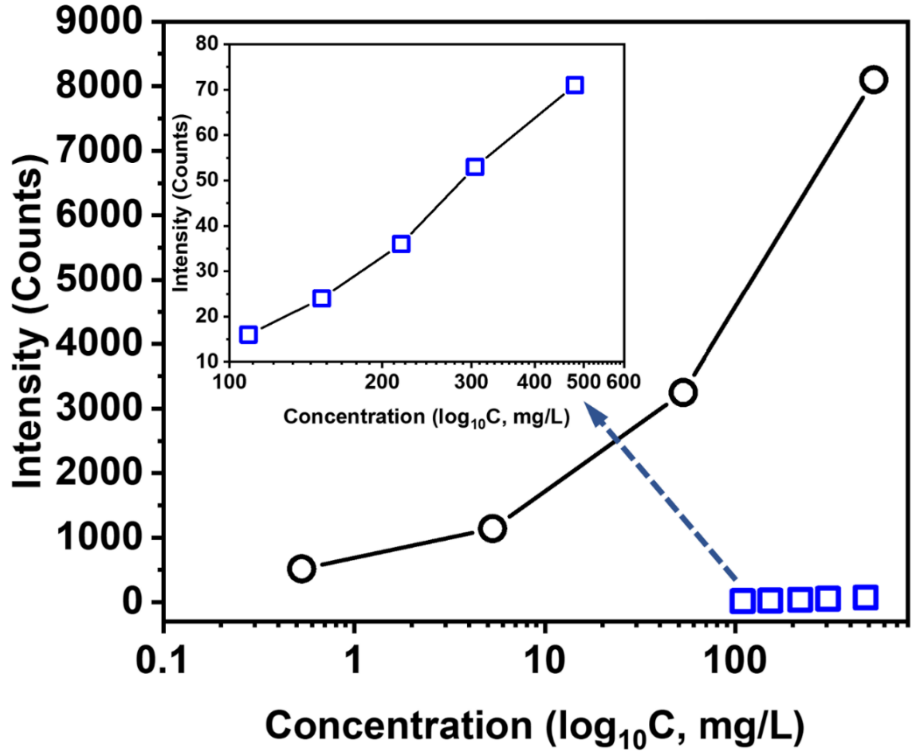

Fig. S3. Raman signal intensity as a function of the toluene/water solution concentration measured using a SERS hollow fiber (empty circles, black) and measured directly using a conventional Raman scheme (empty squares, blue).

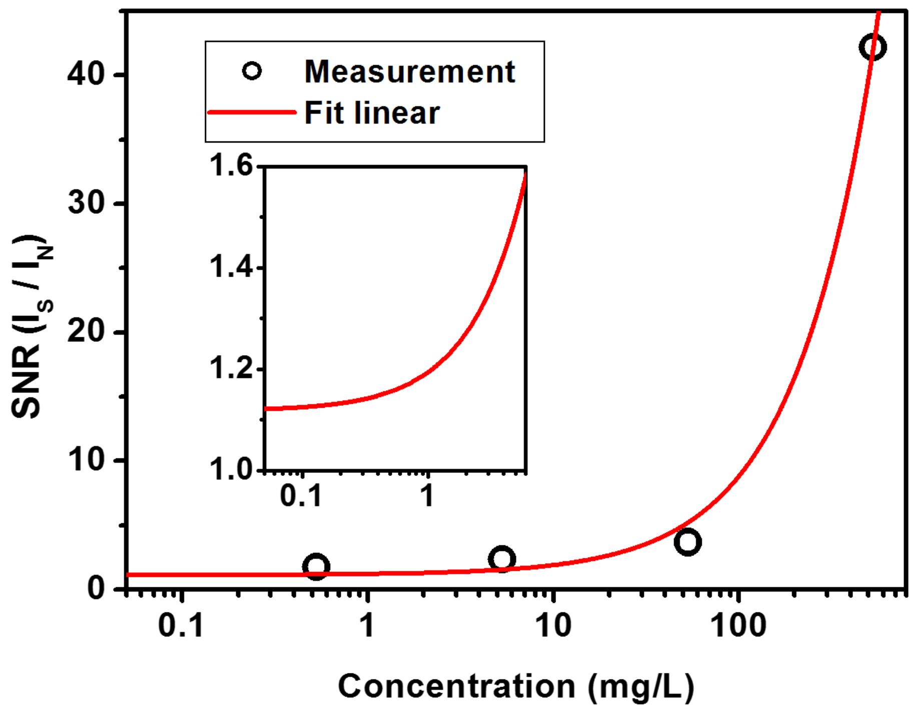

Fig. S4. Plot of the signal-to-noise ratio (SNR) of the measured Raman signal at 1020 cm^{-1} as a function of solution concentration and linear fitting of the measurement data.
